# What do complementary and alternative medicines mean to UK dairy farmers and how do they use them?

**DOI:** 10.3389/fvets.2025.1504777

**Published:** 2025-02-26

**Authors:** Kayleigh M. Crouch, Helen Cramer, Gwen M. Rees, Debbie Sharp, David C. Barrett, Christie Cabral

**Affiliations:** ^1^Bristol Veterinary School, University of Bristol, Bristol, United Kingdom; ^2^Centre for Academic Primary Care, Bristol Medical School, Bristol, United Kingdom; ^3^School of Veterinary Science, Aberystwyth Univeristy, Aberystwyth, Ceredigion, United Kingdom

**Keywords:** complementary and alternative medicine, herd health management, holistic approach, dairy cows, qualitative research

## Abstract

**Background:**

Complementary and alternative medicine (CAM) is used by some farmers to support herd health management practices. There is concern by a large majority of the veterinary community, who consider CAM to be counter to evidence-based practice. Little is known about what and how CAM is used on farms, and it is not clear which products or practices are encompassed by what farmers consider to be CAM. This paper reports on a study exploring the use of CAM on dairy farms in the UK.

**Methods:**

Twenty farms with a range of management systems and herd sizes were recruited. Interviews were conducted with 24 farmers via face-to-face, telephone or videoconferencing modalities necessitated by the Covid-19 movement restrictions. 16 farms were visited to collect observational data using ethnographic fieldnotes and photographs. Interviews were conducted using topic guides and explored participants’ experience of CAM and potential influence on antibiotic use. Interviews were audio recorded, transcribed, and thematically analysed using NViVo software.

**Results:**

A range of views and conceptualisation of CAM was identified among the participating dairy farmers. CAM was not usually seen as one particular product or health management tool but encompassed a range of health management strategies and philosophies. Results indicated that some farmers explore and engage with a range of complementary and alternative medicines and approaches to animal health on dairy farms. Some farmers considered food products, shop bought products, environmental enrichment, in-depth animal observations and technology to form part of their CAM approach. Farmers associated CAM with holistic health management and animal welfare. CAM formed part of a wider ethos regarding holistic farming and land use and was sometimes used to support them in avoiding overuse of antibiotics.

**Discussion:**

Farmers use CAM, and their conceptualisation of it is complex. Several resources and stakeholders were consulted by farmers to understand CAM and conventional medicine. Farmers interest in CAM warrants further consideration. This may support dairy farmers to reduce antimicrobial use responsibly, with veterinary support.

## 1 Introduction

Complementary and alternative medicine (CAM) is used by some farmers to support herd health management practices (1–3). This is viewed with concern by a majority in the veterinary community, who consider CAM to be counter to evidence-based practice. Little is known about what and how CAM is used on farms, and it is not clear which products or practices are encompassed by what farmers consider to be CAM (4–7, 67). This paper reports on a study exploring the use of CAM on dairy farms in the UK. Throughout the rest of this paper, the colloquial term ‘vet’ is used for veterinary surgeons to improve readability and to reflect the term most used by the participants in this research.

The controversy around the use of CAM is rooted in the limited evidence base for its efficacy, where the performance of an intervention under ideal and controlled circumstances is measured (8). The Royal College of Veterinary Surgeons (RCVS), the professional regulatory body for veterinarians, issued a 2017 position statement on CAM, explicitly stating that they do not endorse the use of CAM where there is no scientific evidence of efficacy (9). The statement focuses on homoeopathic practices, largely ignoring the much wider range of CAM products and practices but creating the impression that all CAM may lack an evidence base. Trials of homoeopathic remedies have found that the effect is similar to that of the placebo comparison (10). Taking a side-step from considering the efficacy of CAM research, instead, it is considered here how CAM might assist farmers in managing the health of dairy cows and the influence it might have on antibiotic use. Multiple systematic reviews and research studies highlight the limited evidence base for CAM efficacy, and that there is limited evidence of efficacy for their use currently (11–13). However, some conventional medicines incorporate active ingredients and properties that are derived and extracted from herbs (14–16, 64).

CAM is often presented as an umbrella term that encompasses a variety of different practices. In 2007, the Department for Environment, Food and Rural Affairs (DEFRA) outlined that growing areas of CAM are used in animal health management, including chiropractic, osteopathy, physiotherapy, homoeopathy, aromatherapy, and acupuncture (56). Memon et al., (17) explored the taught content relating to CAM use within veterinary curriculums in America. They found that there was a consensus amongst the colleges that CAM modalities including acupuncture, veterinary manipulative therapy, integrative nutrition, physical rehabilitation and sports medicine, and herbal therapy should be included within taught content. They suggested that including such practices within veterinary education may enhance instructional honesty regarding uncertainties in the field, whilst also producing an openness to new ideas that characterise scientific methods. Bergh et al. (18) characterised CAM as a term encompassing a range of therapies with varying theories and practices identified including aromatherapy, homoeopathy, neural therapy, music therapy, and vibration therapy. This systematic review by Bergh et al. (18) found that 24 complementary and alternative veterinary therapies did not have sufficient scientific evidence to draw clear conclusions regarding their clinical effect. However, Duval et al. (58) suggested that CAM was often used as a means of disease prevention rather than control, so further understanding of attitudes towards CAM would be of value. Tonelli and Callahan (19) also argued that CAM cannot be evidence based. This is because many CAM approaches are centred around non-measurable but perceptible aspects of health and disease within the context of an individual. Instead, Tonelli and Callahan (19) suggest that more complete descriptions and defence of alternative epistemic methods and tools of CAM disciplines are required, rather than insisting that CAM should be evidence based.

The controversy surrounding CAM, especially after the RCVS position statement in 2017, emphasised the need for specific CAM practices to be evaluated separately and avoid conflating the evidence for one type (e.g., homoeopathy) being taken as indicative of the evidence for everything termed as CAM. The BVA, the largest membership community for the UK veterinary profession, released a policy statement in 2018 aiming to define CAM as ‘treatments that fall outside of mainstream veterinary care’ (20). The BVA recognises the challenge of grouping ‘complementary’ and ‘alternative’ together, proposing distinctions between the two. ‘Complementary’ involves using non-mainstream practices alongside conventional medicine, whilst ‘alternative’ entails the use of non-mainstream practices instead of conventional medicine (20).

The awareness amongst veterinarians regarding the utilisation of CAM use on dairy farms and how much vets know about this is not well understood. Poizat et al. (3) noted that dairy farmers in France were often more motivated to use CAM than their advisors or vets. In Norway, Hektoen (1) found that organic farmers would exhaust CAM approaches and treatment options before consulting a vet, potentially raising animal welfare concerns. For instance, whilst Gram-negative mastitis infections may self-heal, Gram-positive infections require conventional treatment (63), though not necessarily that of high priority critically important antimicrobials (HP-CIAs) (21). If farmers’ use of CAM has the potential to compromise animal welfare, for example, if they delay consulting a vet for severe mastitis cases, there is a need to understand the CAM management and products used on farms. Hovi and Roderick (22) highlighted the lack of formal and informal training in CAM for farmers and stockpersons. In the absence of such training and potential vet advice, farmers interested in CAM must rely on other sources of knowledge such as self-learning, peer exchange, or pharmacies offering specific CAM services. Hovi and Vaarst (60) noted that homoeopathic pharmacies in the UK mainly served physician homoeopaths, with farm animal homoeopathic practice only recently emerging. Despite operating on the ‘verge of good and bad practice,’ (60, Page 11) some pharmacies provided problematic advice, diagnosing disease without seeing the animal, and suggesting treatments need not be recorded. By law, milk producers must ensure that milk from animals under antibiotic treatment or in the withdrawal period for any medicine does not enter the food chain (23). Failure to observe milk withdrawal periods and practices would violate the UK law, which requires recorded treatments for farmed animals. Discussing such advice with a vet could mitigate these concerns.

Another challenge to be acknowledged here relates to potential contraindications of evidence-based veterinary medicine (EBVM) and some CAM, whereby active substances in herbal medicines for example might interact with active ingredients in prescription veterinary medicines (24). A vet would be unable to prescribe medicines with the consideration of contraindications if they are not informed of CAM approaches being used on farms. More open communication on CAM use between vets and farmers would be essential to mitigate these risks to animal health, farm contracts, and veterinary professional practice. A reported rising interest in CAM amongst the public will see an expectation for vets to responsibly advise clients who wish to use CAM for their animals. In cases where vets are not involved, it is anticipated that animal owners will seek information from other sources, causing concerns about limited information and contraindications (25, 26). The ability to advise on contraindications relies on vets being aware of the CAM modalities used for the care of animals. As such, exploring the use of CAM in a veterinary context is warranted.

There may be an association between CAM use and improved animal health management. ‘Homeopathy focuses on the individual and not so much on the disease […]. Consequently, homoeopathic care is based on strong partnerships, first between the farmers and their animals, and also between the veterinarian and the farmer’ – [(27), page 1]. Understanding what facilitates open discussion of CAM approaches between the vet and CAM advisors may be of use to inform vet–farmer communication surrounding CAM in the UK. Rees (28) highlighted the importance of autonomy for UK dairy farmers when making treatment decisions, especially relevant as some CAMs, such as homoeopathic products, do not require vet prescriptions. Close farmer–animal relationships, as indicated by Rénier et al. (27), may contribute to preventive and holistic animal health management approaches such as positive housing arrangements, better animal care, feed, watering, and milking thereby reducing disease risk (29, 30, 66). Exploring whether CAM use positively impacts farmers’ general health management approaches is essential, potentially aiding in early disease detection by thorough herd observation. Organic frameworks endorsing CAM provide guidance on housing environments and cleaning regimes to prevent herd infections (31).

It has therefore been suggested that CAM use may have a positive impact for reasons other than an active biological or physiological effect. CAM benefits in human medicine have been attributed by some to the positive relationships built during extended practitioner–patient interactions (32, 33). The term ‘placebo effects by proxy’ describes situations where clinicians and family members, feeling empowered by a treatment, perceive benefits to those in their care even without direct physiological indications (34). Such effects could influence the farming context, affecting how farmers interact with and perceive their animals’ health. Another ‘placebo by proxy’ effect may be relevant to farming, where a farmer’s positivity for CAM enhances human–animal interactions, potentially eliciting positive responses from dairy cows (35, 59, 61, 62, 65).

This study aimed to investigate how and why farmers use CAM on UK dairy farms, and how it might influence their use of conventional veterinary medicine. In particular, understanding what CAM is to dairy farmers, and how they use it is beneficial for two main reasons. First, there is no widely agreed definition of CAM, so it is useful to understand the range of products and approaches that farmers view as CAM, to support clearer communication on CAM use between stakeholders. Second, understanding how CAM is viewed and used by dairy farmers is important to identify whether it influences the use of conventional veterinary medicines, and also to investigate its impact on animal health management practices within UK dairy systems.

## 2 Materials and methods

### 2.1 Study design

This study used a qualitative design combining semi-structured interviews and ethnographic observations. Interviews and observations were both conducted on the majority of farms. However, for a small number, COVID-19 pandemic restrictions prohibited on-farm visits and interviews were conducted online (see Section 2.3 for details).

The ontological approach that this study used was that of relativism, where the researcher accepted that multiple subjective realities exist and have been constructed through the lived experiences of individuals (36, 37). In the context of farming, there may be a variety of backgrounds and experiences of farm personnel, who have developed their own understanding of effective herd health management, which this research aimed to elucidate.

The epistemological approach utilised was epistemic subjectivism, whereby reality was constructed between the researcher and those whose perceptions, values, and attitudes we were interested in understanding (38). In doing so, the researcher accepted that there are likely to be multiple realities amongst participants, and their views are constructed by a range of experiences specific to them (39).

### 2.2 Farm selection and recruitment methods

Purposive sampling was used. The goal was to capture a diverse sample of farmers, recruiting participants with varied herd sizes, management styles, locations, production contracts, and accreditation schemes (RSPCA, Red Tractor, Organic). A heterogeneous sample included key decision-makers and stockpersons on farms. A recruitment poster was circulated on social media and through professional networks. Gatekeepers from a range of industry organisations also supported recruitment through the poster. Farmers were initially sought with the caption ‘calling organic dairy farmers’ in the South West, broadening later to ‘calling dairy farmers’ for wider representation. The iterative approach, leveraging social media and gatekeepers, resulted in participants from conventional and non-conventional systems across diverse regions.

### 2.3 Data collection

A total of 20 farms participated, with 17 visited in person. Due to COVID-19 restrictions, some interviews had to be conducted remotely using University of Bristol-approved software (Zoom Video Communications Software) or telephone. The remaining interviews occurred in person on participant farms, ranging from 30 to 90 min in length. Most interviews were conducted with individuals, but some were with pairs or trios of farmers. Further details are included in Table 1.

**Table 1:** Farming systems, herd sizes, and participant’s roles.

		Number of participants
Farm system	Organic	15
	Conventional	9
Herd size (n = dairy cows)	<100	8
	100–199	8
	>200	8
Interviewee role	Owner of farm/dairy/calf unit	5
	Manager of farm/dairy/calf unit	16
	Head herd-person/milker	3
	Total sample	24

The sampling approach was guided by iterative and ongoing data analysis and observing when data saturation was achieved. Data saturation is the point of data collection where the analysis is no longer developing, and no new data are being collected (57). Based on previous research that used interviews to understand what drives farmers’ use of CAM in different cultural contexts, e.g., Spain (2), Norway (1), and France (3), the phenomena of interest were predicted to consist of a wide variety of views, experiences, and practices and as such the information power relative to the research aims may be low (40). Information power refers to the model designed by Malterud et al. (40) which states that the more information the sample holds, relevant to that study, the fewer participants need to be interviewed. However, this sample size was also informed by continued data analysis, paying attention to the contribution and variety of new knowledge to the dataset, rather than solely on the number of participants. Data saturation in previous research of this kind ranged from 18 to 56 interview participants. The participants recruited were also constrained by the practical means (time, location, and financial means) of the project (1–3). A total of 9 female and 15 male dairy farmers participated in this study.

Topic guides (see Supplementary Material) were used to ensure comparability across the farmer interviews and ensure that the questions asked were appropriate for gaining insights relating to the research aims. The topic guide included: (1) the farmer’s CAM use and general awareness of CAM use within the farming community, (2) the farmer’s application of CAM on the farm and whom they might seek support from, (3) the farmer’s perceived outcomes of using CAM (benefits and risks), and (4) the potential practice-based benefits of using CAM. The semi-structured interviews were conducted which allowed participants and the researcher to diverge and pursue an idea or response in further detail where necessary (41). Following the methodology advocated by Vickers and Zollman (42), the researcher adopted an open approach to eliciting the specific practices, products, and approaches considered CAM by participants in this study. Importantly, the study avoided imposing a rigid theoretical definition of CAM, aligning with an explorative research approach and the absence of a widely agreed-upon definition in the field.

Ethnographic observations were conducted at 17 farms with diverse characteristics (43), and were conducted at the same time, on the same farms, as the semi-structured interviews. The researcher observed general characteristics, herd management, and CAM practices, supplemented by short ethnographic interviews. Fieldnotes (44) were recorded in the researcher’s phone notes function for practicality during farm walks, which allowed the capture of additional information. In certain instances, conversations are recorded with farmers’ permission using an encrypted audio recorder, especially in fruitful settings such as medicine storage areas. This on-site data collection informed the researcher about current CAM practices on dairy farms, guiding subsequent research phases. Fieldnotes served a dual purpose by aiding researcher reflexivity and guiding subsequent research phases. Due to COVID-19 restrictions, it was not possible to visit all farms in person. Participants were encouraged to share additional information via email and this was incorporated into the fieldnotes and analysed in the following section. Types of information shared included a list of CAM books, pictures of specific CAM products, and examples of Obsalim® cards used to assist farmers in monitoring early signs of disease (54).

Semi-structured interviews ranged in length from 36 min to 96 min, with an overall average length of 62 min. Of the 17 farms that were visited, the total length of farm visits ranged from 1 to 4 h. Farms involved in this study were geographically situated between northeast England and mid-Wales. Farm visits and semi-structured interviews all took place between September 2020 and June 2021. Further details of the farmer roles, farm systems, and sizes are shown in Table 1.

### 2.4 Data analysis

Upon completion, each interview underwent manual transcription and anonymisation before being imported into NVivo 12. Transcription was completed manually by an experienced University of Bristol-approved transcription service, and the transcripts were checked for accuracy by the lead researcher (KC). Fieldnotes were transcribed (by KC) and included in data analysis. The study adopted an inductive thematic analysis approach (45, 46) to identify patterns, create codes, and develop themes reflective of the findings. All transcripts and fieldnotes were coded by the lead author (KC) promptly post-interview to inform subsequent semi-structured interviews. A sub-sample of transcripts was read and independently coded by other authors (CC, HC, and GR). The team met several times to discuss and agree on the final codes. The codes were designed to stay closely connected to the data, incorporating terminology and phrases from the transcripts and fieldnotes. This coding process was applied consistently across both interview transcripts and fieldnotes.

The consistent use and iterative updating of codes facilitated ongoing data import and analysis, ensuring the incorporation of new information. The fieldnotes were analysed concurrently with the interview transcripts, contributing to the overall thematic development of the results. This approach enriched the analysis by capturing details not initially addressed in interviews.

### 2.5 Research team positionality

The same researcher conducted the interviews and farm visits (KC). KC was a PhD student at the University of Bristol and introduced themselves as such to participants. KC has a background in Animal Behaviour and Welfare (BSc and MRes). All participants were aware that the project was run through Bristol Veterinary School, but that the interviewer/researcher did not have clinical veterinary training. Other members of the supervisory team were from multiple disciplines including population health sciences in human medicine and veterinary science, including a recognised specialist in bovine health management. This provided a balanced and informed range of perspectives on the research topic.

## 3 Results

The analysis produced a list and rationale for products and approaches that were considered to be CAM by these farmers and three overarching themes: ‘How farmers conceptualized CAM’, ‘CAM use was viewed as an act of care’, and ‘Farming approaches associated with CAM use’.

### 3.1 The range of products and approaches identified as CAM used by farmers

Farmers considered a range of products and approaches to be included under the umbrella term ‘complementary and alternative therapies’ (Table 2). These products and approaches were described as tools that contribute to overall herd health management and included homoeopathy, herbal remedies, distant healing, food products used for ingestion or external rubs, observation methods, shop-bought products, environmental enrichment, and some conventional medicines. This range of products was also used for various health management needs and is outlined in Table 2.

**Table 2:** A summary of complementary and alternative approaches as defined by participating farmers, and why they are used.

Complementary and alternative therapy types	Farmer reported reasons for use	Examples include
External herbal rubs	To manage bruising, abdominal discomfort post-calving, mastitis, pneumonia, digestive function, worm control, and back soreness.	Uddermint Golden balm Comfrey Calendula Avena Masticare oil
Herbal essential oils	To manage metritis, calming cows in stressful surroundings, digestive function, and mastitis control.	Lavender oil Citronella and lavender Avena masticare oil
Food products ingested	To manage gut health and support digestive functioning.	Kefir Apple cider vinegar Sea solids Garlic puree Garlic
Products used as internal washouts	To manage retained placentas, foetal membranes, and endometritis.	Coffee UTREsept® Apple cider vinegar
Products used as external rubs	To manage wounds and healing sores.	Manuka honey Pine resin Coconut oil Peanut oil
Products used as external wash and applied externally	Used as a fly repellent, stress management, digital dermatitis, mastitis prevention, numbing sores, and wound barriers, to manage bloat.	Epsom salts Listerine Germolene Skinsosoft and tea ‘Stinky stuff’ (containing *nigella sativa*, *cocos nucifera*, butyrospermum, parkii butter, *euphorbia cerifera* cera wax)
Environmental enrichment	Providing occupational activities, maintaining healthy coats, improved quality of life for animals, reducing stress at handling, and maintaining a well-functioning immune system.	Occupational enrichment Cow brushes Positive human–animal interactions (e.g.*, calm talking and scratching*)
Observational tools	Monitoring animal health and disease, monitor animal behaviour, udder health during milking, and preventing mastitis. Furthermore, sometimes used to inform farmers of which CAM approaches might be most appropriate.	Obsalim Technology Robotic milking statistics Constitutional element of homoeopathy
Proximal plant placing	Ringworm.	Holly bush
Homoeopathic products	To manage a range of health conditions, including mastitis, delayed development, lethargic behaviour, animal stress, arthritis and joint pain, urogenital problems, pain, bruising, throat soreness, fear, shock, cuts and abrasions, high fever, swelling, septicaemia, necrosis, milk fever, digestive disturbances, coughing, pneumonia in calves, to maintain good health, bloat, reproduction/fertility, enteritis, ease of calving and weaning, prolonged wound bleeding, skin irritation, post caesarean section recovery, and digital foot health (*further details available in* Supplementary Material).	Constitutional element of homoeopathy Premade kits Single remedies Bespoke remedies for farm/animals
Bioresonance products	Providing animal or herd-specific products for a variety of animal health challenges, e.g., ringworm, nausea/vomiting for cows in calf, stress, and improving milk quality.	Bioresonance (a machine used to transmit information through electromagnetic waves to provide treatments to restore cellular balance, stimulate tissue regeneration, and support self-healing) services through pharmacies
Distant healing	To heal specific animals or herds with positive vibrations by people in locations that are viewed to be conducive for effective dowsing.	Sending vibrations to heal animals using pictures/locations on maps

#### 3.1.1 Herbal products

A wide range of herbal approaches was described by farmers and was seen to contribute to their CAM approach. Several farmers had explored the use of herbal remedies as a result of on-farm sessions where they were taught how to make herbal salves and tinctures by a herbalist.

*“But she [herbalist] actually did make salves with us and things like that [Participant 15]. Showed us how to make herbal remedies and things [Participant 16]. Tinctures. It was a bit more hands-on rather than just theoretical [Participant 16].”* - Participants 15 and 16 (Organic dairy farmers) [Interview excerpt].

Stockholm Tar® is made of Swedish pine tar and is applied topically to areas of concern. Several farmers stored Stockholm Tar® on the farm to reportedly manage mastitis and foul of the foot. One farmer used Stockholm Tar® to support foot health.

*“foul of the foot is a big issue on farms so Stockholm Tar… they all take more time [than injectable antibiotics].”* - Participant 10 (Organic dairy farmer) [Interview excerpt].

A product called UTREsept® was used by one farmer as a uterine washout to support cleansing and manage retained placentas. This is a readily prepared product purchased online that contains yucca plant, citric acid, and orange.

*“This is the uterine wash out which has yukka in it to make it go soapy and then the citric acid and orange gets rid of the bugs or helps with cleansing and retained placentas”* - Participant 24 (Organic dairy farmer) [Fieldnotes excerpt].

Nine farmers had the experience of using essential oils to support their herd health, some as a result of a Replacement of Contentious Inputs in Organic Farming Systems (RELACS) project run by the Soil Association (SA). This project investigates the effect of using essential oils on disease to reduce the need for antimicrobial use (AMU). One farmer was motivated to use essential oils to reduce stress or problems with lactation as a result of experiential evidence and media.

*“I think, with essential oils, … I had a little look online at them, to see which ones I can buy and see which ones have the benefit from it. I think, especially in some of the equine magazines and publications, in there there’s been quite a lot of people using them… I’ve used essential oils for myself in the past, …they are very good and can be very calming and help, especially for aiding sleep… And lactation, …I put citronella, I put lavender, … I think there were four or five that I put into that spray combination, for them.”* - Participant 22 (Organic dairy farmer) [Interview excerpt].

##### 3.1.1.1 Herbal shop-bought products

Farmers frequently discussed the use of products that they obtained from [online] retailers. For example, farmers used Stockholm Tar® to manage discomfort.

*“That [Stockholm foot tar] is used to help with hard or blown udders … We had one with a burst abscess and so smeared some of that all over to help keep the flies off too.”* – Participant 10 (Organic dairy farmer) [Fieldnotes excerpt].

Another shop-bought product used by one farmer was Avena Masticare®, an udder care and teat oil containing sunflower, lavender, eucalyptus, geranium, and rose essential oils. This was used alongside other essential oils and water treatments to manage mastitis.

*“So, we have been using that [Masitcare®] like I say for a long, long time and we do find that does help with some swellings and aids cows.”* - Participant 14 (Organic dairy farmer) [Interview excerpt].

##### 3.1.1.2 ‘Everybody uses Uddermint®’

Every participating farmer either had experience of or used, a herbal product called Uddermint® at the time of the interview. This is because the use of Uddermint® was seen as a normal practice within farming communities, and farmers all know other farmers who used it and therefore had faith in it to be a reliable CAM product.

*“I think most people do use it [Uddermint]… most of my farming friends would use it … I guess it’s so long ago that we started using it… I think it’s been around so long, not so many people talk about it anymore, so I think it’s a given that nearly everybody uses it…”*– Participant 12 (Conventional dairy farmer) [Interview excerpt].

and

*“Uddermint® has always been around. It’s been around for a long time. It was always in the milking parlours. It’s sold by everybody. It seems to be the market leader and everybody knows it. Everyone knows what it is and what it does…”* - Participant 10 (Organic dairy farmer) [Interview excerpt].

The storage or location of certain products varied depending on whether farmers considered them to be CAM or conventional medicines. For example, Uddermint® was not considered medicine and so it was not deemed necessary to store it in a medicine cabinet the way conventional medicines are stored. Uddermint® was commonly found hung up in milking parlours to be used during milking.

**Figure 1:**
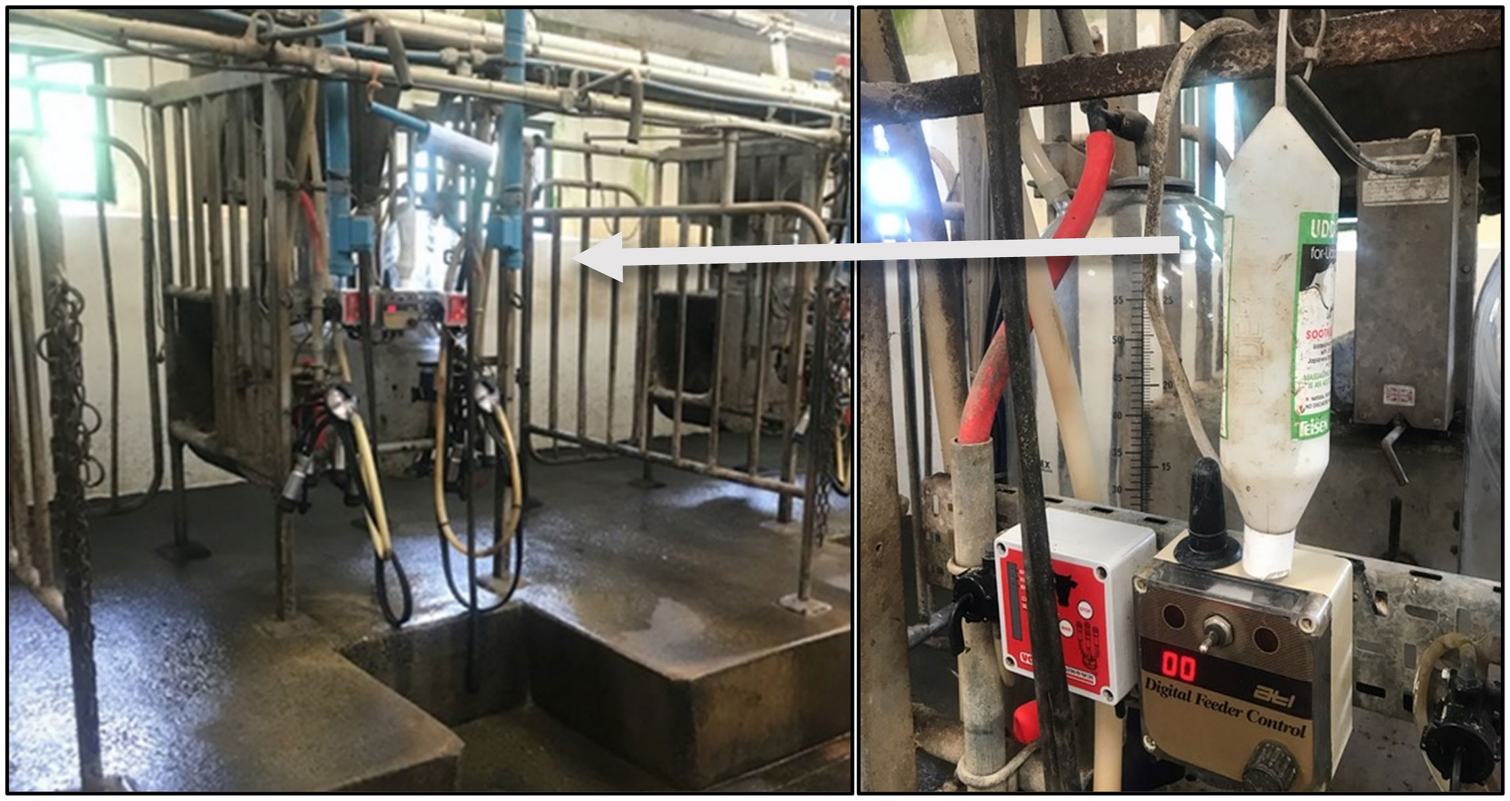
Uddermint® hung up in the milking parlor on a UK dairy farm.

*“Uddermint was obviously hung up in the parlour [see* Figure 1*], and we have a big tube with a squeezer on the bottom, and then, if you need it, you just squeeze it at the bottom, and then it’s hung on the parlour wall – ‘that should not be in the parlour because it’s like a medicine’ [farm auditor]. I said, ‘that’s ridiculous’… I sort of pooh-pooh-ed it and said I’ll move it out and never did, and it was a long time ago, but it seemed ridiculous because it’s not really a medicine at all, so… It worked – well, it’s not POM [prescription only medicine] is it?”*-Participant 12 (Conventional dairy farmer) [Interview excerpt].

#### 3.1.2 Food products used as external rubs and to be ingested

CAM was used for wounds and clinical conditions when conventional medicines were not considered appropriate methods to manage discomfort. This included the use of products such as [Manuka] honey or conventional antiseptic Sudocreme® on cuts, open wounds, and displaced abomasum; caffeine pumps for displaced abomasum; and peanut oil for cows with bloating.

*“I mean the Manuka honey, absolutely brilliant. We’ve got some over in the calf shed but we are not actually using it. We had it in case we needed it because I said about how good it was for wounds and things like that and obviously if you have got calves bouncing around in pens they are going to get cuts and bruises and things.”* – Participant 2 (Conventional dairy farmer) [Interview excerpt].

and

*“Bloat is another thing, you can give animals – if you give them peanut oil and stuff, so we have done that in the past. It’s amazing really how stuff like that, cheap stuff off the supermarket shelf quite often can be the thing yes.”* – Participant 19 (Organic dairy farmer) [Interview excerpt].

#### 3.1.3 Environmental enrichment

Approximately a third of farmers considered different forms of environmental enrichment to form part of their CAM approach. One farmer explained that environmental enrichment in the form of traffic cones was used as a distraction for calves to prevent them sucking other calf’s navels. This was because they wanted to prevent bacterial infections in calf navels and so introduced something to occupy them when they were not eating.

*“…most farms are conventional and they are using antibiotics …but hopefully we are starting to introduce things here with enrichment and cow welfare that I would class as complimentary… when you wean a calf, so you are stopping feeding it its milk, if it can still see everybody else getting milk then suck reflex is still there, so because they are not sucking the milk they’ll then look for something else they can suck, which usually is another calf’s navel, so you are then going to introduce bacteria and things back into the navel that you really do not want, so you want them to be able to do something to keep their mind off of what’s going on. We used to use a traffic cone, one of the great big motorway traffic cones, …because they can knock it and it bounces back. …it’s just something that’s occupying them while everything else is going on, so it’s a distraction.”* - Participant 2 (Conventional dairy farmer) [Interview excerpt].

This overview of what farmers consider to be CAM demonstrates that a wide breadth of practices, production, and healthcare approaches are used to support them in herd health management.

#### 3.1.4 Observational tools

Observations of animal behaviour, characters, and signs of health were commonly considered part of a farmer’s CAM approach and comprised strategies to support herd health management. One example included Obsalim®, which is an observational tool used to link indicators of health with nutrition and health maintenance (54). Obsalim® is run by a homoeopathic vet, a vet who is involved in supporting farmers moving to contracts that require them to produce milk without antibiotics (PWAB), and a pharmacist who works closely with farmers to provide homoeopathic services and advice. Obsalim® was used by one farmer who regularly engages with Obsalim® groups.

*“…we have Obsalim®, which I think is great because if their nutrition is not right then no medicines or alternatives are going to work are they. You need to get the basics right first… I had a homeopathic vet here, he is the guy that’s now set this up… but I also run a group as well with farmers and we meet once a month … There’s probably eight of us. We meet together and we go through each farm, do an Obsalim® diagnosis on the farm and then all decide what should happen. What the solution should be.”* - Participant 24 (Organic dairy farmer) [Interview excerpt].

Technology was another tool used by farmers to monitor animal health. Technology would alert farmers when a cow requires attention. MooMonitors® (collars with a device that monitors neck movements via heat-related activity to indicate the frequency of rumination, resting, feeding, and restlessness) for example, were used by farmers to alert them when an animal is behaving in a way that is indicative of a decline in health, particularly in relation to mastitis.

*“We use moo meters to monitor heat detection, but that also does rumination. So, what we are finding is if a cow drops in rumination we’ll get an alert on the phone before we’ll see it in the udder, if she has mastitis. So, we are gaining time and I think that’s essential in fighting mastitis. The sooner you can get in there the better.”* - Participant 14 (Organic dairy farmer) [Interview excerpt].

When asked whether they considered technology as part of their CAM approach, the farmer who used MooMonitors® suggested that it was. This was because it enables farmers to detect clinical signs sooner, rather than waiting for more severe clinical signs to develop.

#### 3.1.5 Proximal plant placement

One method mentioned by [three] farmers for controlling ringworm was hanging holly in a cowshed. Despite not understanding the mechanisms by which this would work to control ringworm, this farmer asserted that it was effective based on their own experience. Interestingly, this was described as a homoeopathic approach.

*“…something we have used which is the holly, … we used to get a bit of ringworm, and that was in a book – I think it was a dairy book – it was about using male holly and hanging it up in the shed, helps with ringworm and we did that and it completely works… It absolutely works and we used it… it’s got to be the male… Obviously, that’s the homeopathy treatment – you just hang it up. The animals cannot touch it or anything. There’s definitely something in it. The thing is – it’s hard to work out because there’s not that much expertise – you have to work it out yourself.”*-Participant 12 (Conventional dairy farmer) [Interview excerpt].

#### 3.1.6 Homoeopathy

Eight participating farmers used homoeopathy at the time of the interview and valued the ability to provide complementary care to animals alongside conventional treatments. Homoeopathy was underpinned by an alternative philosophy that was used in a complementary manner alongside conventional veterinary medicines. Multiple farmers used homoeopathy to fill a void during the time between identifying a clinical sign and a vet visit.

*“I mean, we reach for it and just get on with it quickly because it’s something that’s there and you can do it and then even if we’d call a vet or something we might just say ‘right, well, let us give her this’. The thing with homeopathy is we have got a cupboard full of remedies and they are very specific for very specific purposes and actually you have to get things quite… you have got to be quite accurate.”* - Participant 4 (Organic dairy farmer)[Interview excerpt].

A few farmers observed the animals’ personalities and behaviour alongside signs of health to identify which remedy was most appropriate to use. One farmer explained that homoeopathy promotes matching the correct remedy to a specific animal rather than for a particular disease or clinical sign.

*“… its [homeopathy] not just a pill for an ill. You cannot just say ‘Oh it’s Staph aureus mastitis, let us give this remedy’. It does not work like that sadly. It would be so much easier if it did. So, you have to take into account her mastitis, obviously. So if it’s really swollen quarter that’s red, throbbing and perhaps a bit of milk dripping out and the cow herself has sort of barged into the parlour and she’s a bit ‘raaaaaa!’, so taking her behaviour into account as well, you would know that remedy because you’d think, ‘Yes, three good things about her, preferably four good things about her points to [remedy].”* – Participant 24 (Organic dairy farmer) [Interview excerpt].

Some farmers focussed on supporting animals to stimulate their own immune response/healing ability through homoeopathy.

*“…I’ve never liked giving drugs and the drugs with side effects… I have not ever liked that because I’ve always thought ‘There must be a better way to help a body get better’, rather than just trying to treat what the symptom is and I’ve always felt it’s wrong to do that. I think bodies should be stimulated into doing the right thing.”* – Participant 24 (Organic dairy farmer) [Interview excerpt].

**Figure 2:**
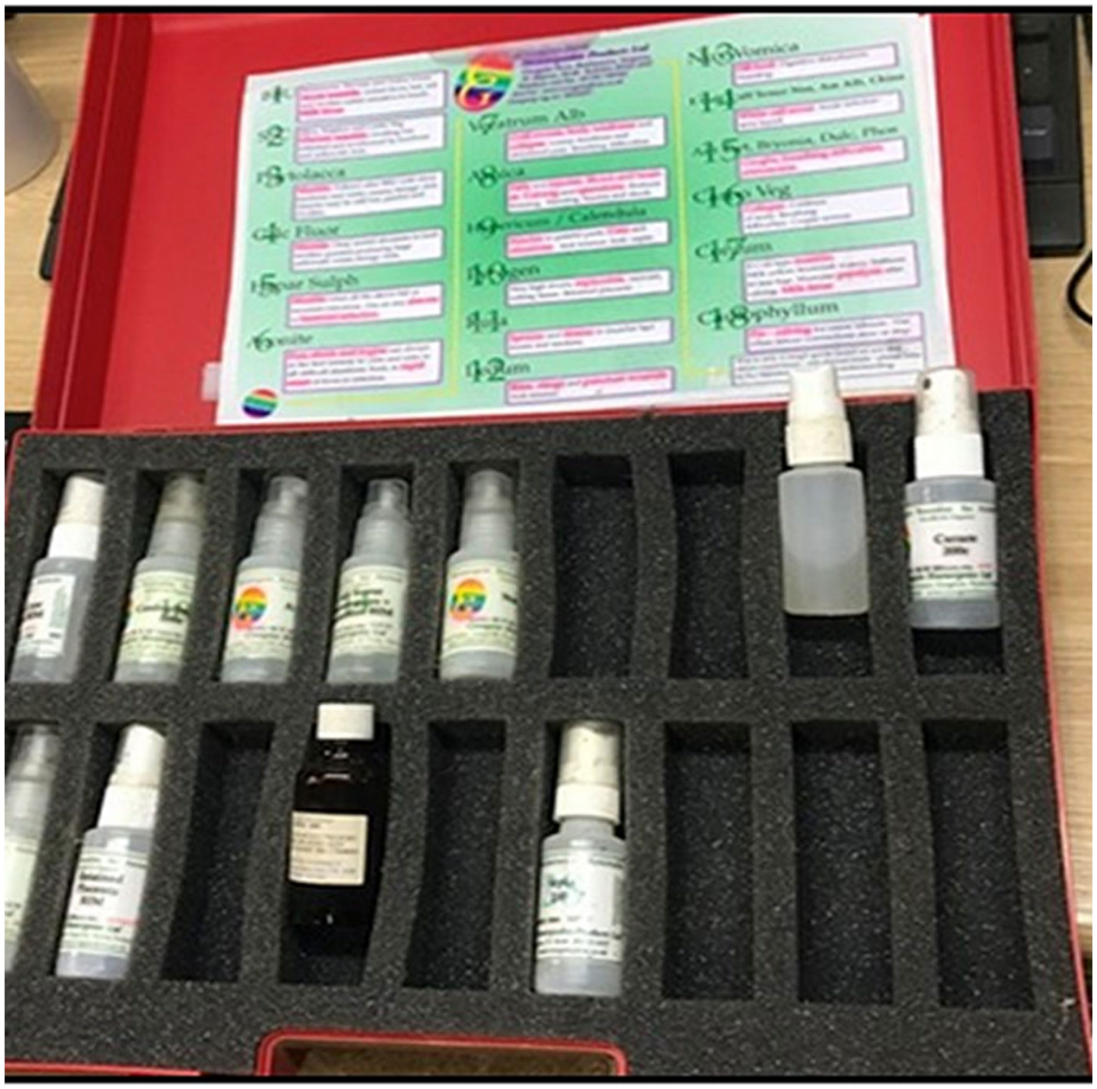
Photograph of a remedy kit and accompanying treatment advice handout.

Homeopathic toolkits were observed during farm visits provided by three different pharmacies. Homoeopathic toolkits are homoeopathic preparations stored in a case, with accompanying descriptions of each preparation and when they should be used. A homoeopathy kit situated in the farm office (Figure 2) was acknowledged by the farmer.

*“There’s lots of bits kicking around in here [farm office] and there will be some more lingering around outside… This is the toolkit […] tells you the bottle and what it can be used for, and we have had other toolkits which will have the problem and then the possible remedies to try.”* – Participant 11 (Organic dairy farmer) [Fieldnotes excerpt].

The constitutional element of homoeopathy was a complementary observation tool used alongside homoeopathy to identify an appropriate given remedy. This was a method described as supporting observations of an animal as a whole being rather than focusing specifically on the clinical signs of the disease in isolation. One farmer described this as an acute observational method which is important for successful health management.

*“I think homeopathy is quite a subtle approach and it does involve some pretty acute observation I think, and I think that’s an important part of homeopathy and I think [Homeopathy at Wellie Level (HAWL) advisor] would agree with that… You have what they call the constitutional element of homeopathy which is where you actually take into account the animal, not just the ailment.”* - Participant 4 (Organic dairy farmer) [Interview excerpt].

This approach encouraged farmers to observe and identify specific animals who are more prone to health concerns and would therefore apply homoeopathy to prevent disease. Some farmers would record which animals are most prone to a specific disease or condition and predict the likelihood of this reoccurring, rather than administering homoeopathic remedies on a case-by-case basis.

*“… because you can go to the next level which is about trying to decide the type of animal rather than treating a particular case, if you like. So, that’s more looking at constitutional remedies is what it’s called, so it’s looking at ‘so this animal seems to always have this problem and it might be nothing serious-serious but it might just be that she goes a little bit lame sometimes’ so you can go ‘oh, well, why is that happening?”* – Participant 11 (Organic dairy farmer) [Interview excerpt].

#### 3.1.7 Bioresonance

Farmers who used homoeopathy also often used services provided by pharmacies with the goal of identifying appropriate remedies for specific animals. Three farmers used a service named Bioresonance, which involved sending samples of hair or feathers to a pharmacy, for them to run tests. These tests involved measuring the frequency of energy wavelengths to inform what condition is present and which remedies should be used to manage it.

*“… [pharmacy name] do a thing where you can send off feather samples and they have come up with a… it’s called bio resonance, I think, and they’ll come up with a remedy which is basically made to balance what’s wrong… so you can actually come up with a made-for-you type of product. They can do that with hair samples as well with cattle”* - Participant 11 (Organic dairy farmer) [Interview excerpt].

#### 3.1.8 Distant healing

Three farmers from the same farm used distant healing which involved communicating with someone who would dowse [a form of divination to seek knowledge of the future through supernatural means] from Australia in a particular location that provided correct energetic waterways. The farmers contacted these individuals to heal the farm when they first moved in. The process involved dowsing for a specific remedy using a map with the location of the farm and then sending vibrations via their fingers and appropriate remedies created using a Sulis machine (a machine used to make energetic remedies and combination remedies that simulate homoeopathic remedies).

*“…‘Heal with Ease’ in Australia and we’d done dowsing with this [distant healer] to dowse where you put the power towers in the first place – you have got to have them over-crossing energetic waterways sort of thing – and they were into dowsing, homeopathy and radionics. It was really spooky… they do, how can I say, ‘distant healing’ – and we have had them heal the farm. Do not tell Dad this! They literally do it off a map. The new farm we took on was a little bit ‘yuck’ so anyway we feel that it definitely works. They’ll even do people as well at a distance… It involves dowsing for homeopathic remedy and then sending it on Sulis machine. Have you seen a Sulis machine? … A Sulis machine sends the vibration to you, so it’s all about vibration… And your date of birth and yes, they heal like that. [Participant 15]. They use their fingers or something…. The resistance of their fingers [Participant 16].”* - Participants 15 and 16 (Organic dairy farmers) [Interview excerpt].

The following sections describe three main themes identified, which reflect how farmers use CAM and the way it was described to align with their goals as dairy farmers.

### 3.2 How farmers conceptualised CAM

There were four key ideas of what CAM was that came through in the interviews. CAM is a holistic system approach; CAM is a logical approach; CAM is magic; and CAM is a natural approach.

For some farmers, CAM was a whole approach and philosophy of farming and the environment rather than just some alternative products to conventional medicines that they might use. CAM approaches were described as health management tools that enabled farmers to trust their own instincts and their ability to maintain a healthy herd. Farmers introduced nutrition, general health observations, breeding/consideration of heritable health traits, and farm design as part of their disease prevention methods.

*“It’s all about having a farming system which enables good health in cows which goes from what we are feeding which depends on what there is in the soil, nutrition and what traits we are breeding forward to remain landscape tuned and really it all comes back to wellbeing”* - Participant 21 (Organic dairy farmer) [Fieldnotes excerpt].

There was a general consensus that CAM is something that influences the actions of those who are responsible for the care of dairy animals and encourages a greater understanding of factors that contribute to poor health. CAM approaches were thought to be useful to calm both animals and stockpeople during handling or moving the cows. They explained that there was a wider influence on animal health including immune function, heritable health traits, and yield capacity of an individual cow.

*“But the other side of it, arnica [homeopathic product] and trying to calm cows down but then part of that is trying to calm people when they are moving cows, dealing with cows, stopping injuries, …part of that is looking at underlying health like the why are those cows sick, lower immunity, what family lines there are, what yield can they stand…”* – Participant 10 (Organic dairy farmer) [Interview excerpt].

Some farmers referred to different types of complementary and alternative approaches as something rational/logical and used by other farmers or professionals within the farmer community whom they consider to be ‘sensible’ or ‘logical’. Some farmers saw some CAM approaches as logical because of their association with conventional veterinary medicine, particularly in the case of herbal medicines.

*“Willow for instance has aspirin in it doesn’t it?”* - Participant 23 (Organic dairy farmer) [Interview excerpt].

and

*“Sometimes they work, sometimes they do not. There must be some logic to it somewhere.”* – Participant 6 (Conventional dairy farmer) [Interview excerpt].

Some farmers used terminology associated with the concept of magic with specific CAM practices, and in particular when referring to homoeopathy. The outcomes of CAM practices were sometimes also described as miraculous. Some farmers acknowledged that some CAM approaches were difficult to understand or explain, but believed they did work to support them in managing animal health on farms.

*“Realistically you want to get cows stripped out …, you want to take the heat out and you want to calm them down… The magic thing.”* – Participant 10 (Organic dairy farmer) [Interview excerpt].

and

*“[Landowner]‘s done one [HAWL course] and he’s… got some potions he brings out every now and then…”* – Participant 18 (Organic dairy farmer) [Interview excerpt].

Farmers often made associations between CAM and nature and referred to CAM practices as a method to provide natural healthcare and/or work alongside nature. Some farmers described using ingredients that were grown in the wild and harvesting particular herbs to prepare their own remedies. Farmers frequently viewed natural CAM approaches as less harmful than conventional medicines and often contrasted the two approaches. Healthcare products that required no milk withhold (‘withdrawal’) or had no risk of taking too high a dose were preferable over conventional veterinary medicines which farmers often felt did impose risk.

*“…a lot of it’s [homeopathic remedies] just using stuff that grows wild, identifying the plants and then preparing stuff and giving it to animals.”* – Participant 4 (Organic dairy farmer) [Interview excerpt].

and

*“…Because I think there’s no withdrawal or you cannot overdose on it [Uddermint®], you can do what you want with it pretty much and you are not going to cause a problem are you…”*– Participant 9 (Conventional dairy farmer) [Interview excerpt].

The reason that technology such as the use of robotic milking was considered CAM was that they work by making the cows’ lives more natural. Robotic milking was used to mimic a more natural milking experience for the cow. This is because clusters are released from the teat when milk letdown is ceased, similar to when calves stop suckling when they are no longer receiving milk.

*“What it will also do is tell you how much each quarter is producing, and if one quarter is not giving anything then the cluster will just come off’…‘it’s more natural that way.”* – Participant 9 – (Conventional dairy farmer) [Fieldnotes excerpt].

### 3.3 CAM use was viewed as an act of care

Farmers described a variety of motivations for using CAM. Most often they desire to do something to support the health of their animals when other approaches are not available or appropriate. Acts of care were presented as a key motivation to use CAM because it provided farmers with a tool to care for animals outside of conventional veterinary medicines. CAM used in this way included a range of treatments and approaches including medicines prescribed by a vet, homoeopathy, foods (manuka honey), herbal treatments (Uddermint®), and some broader CAM approaches including behavioural observations. Farmers report that they utilise tools that they consider to be CAM to provide care for livestock; monitor, prevent, or treat disease, and also satisfy the duty that they feel they have as animal caregivers responsible for looking after dairy cows.

Farmers frequently recalled situations where they used CAM for conditions or ailments that would cause discomfort rather than pain. For example when dairy herds are exposed to flies. Farmers used Stockholm Tar®, a combination of stinky stuff®, Dettol®, and cold tea or Uddermint® to manage this discomfort. Stinky Stuff® was a shop-bought herbal product that is advertised as a natural balm that can soothe irritated skin (see Table 2 for product ingredients).

*“I do spray it on the calves [Stinky stuff, Dettol and cold tea]. I think that really helps them. When there’s lots of flies about, you know, when it’s really hot. It certainly – my husband has often said to me, ‘Your stuff does keep the flies off them.”* – Participant 20 (Organic dairy farmer) [Interview excerpt].

Farmers use CAM to reduce the stress of their herd, by making animals more comfortable, calming them down, and providing an environment that is conducive to animals looking after themselves. CAM has been discussed in the broader context of emotional or physiological stress reduction. This is often described at the herd level whereby products such as homoeopathic remedies or herbal preparations were put into water troughs or sprayed in the cowshed before certain events to prevent handling stress.

*“There are some sort of main remedies that we were taught, like Arnica, Aconite, which is a sort of stressy thing. So I could use those like after calving, for any stressful situations like de-horning, TB testing. So I started off with just the ones that I was able to and I thought ‘Well, this is not going to cause any harm if I get it wrong.’ So that was good” –* Participant 24 (Organic dairy farmer) [Interview excerpt].

Weaning was recognised as a stressful event for livestock by farmers, particularly as it is associated with the separation of calf and cow. Farmers commonly used ignatia to minimise the stress caused by weaning, either in spray form on a mucous membrane or into water troughs.

*“Then there’s another good one that’s called Ignatia which is really good around weaning, so you give it both to the calves and the cows, or the lambs and the ewes, and it just seems to make weaning go significantly easier.”* – Participant 11 (Organic dairy farmer) [Interview excerpt].

CAM was used to alleviate physical stress during difficult calvings. One farmer recalled using homoeopathy to provide comfort and reduce stress to a cow who came into calf at 16 months of age (generally first calving would take place approximately 24–25 months). The farmer could not see that the cow would recover from such a challenging calving at such a young age but felt that they did due to administering aconite and arnica.

*“…Anyway, we had to calve this animal… So, we pulled this calf out and the poor heifer just laid there and I said ‘look, I’m going to go and phone the kennels now’ because they can shoot this heifer – she was obviously so badly damaged by this process and she’s going to be suffering… So, I went and phoned the kennels and I could not get a reply… I said ‘oh, look, we’ll give her arnica, aconite and…’… cut a long story short, I never did get hold of the kennel. She got up and seemed remarkably good… Anyway, she went on and fully recovered amazingly quickly. I would have put a lot of money on the fact that she would never have recovered from that because it was so brutal.”* – Participant 4 (Organic dairy farmer) [Interview excerpt].

Farmers described CAM as something that gives them the ability to do something to care for an animal, as opposed to not providing care at all. It was seen as something that they could use autonomously, with little input from other stakeholders. As such, farmers gained agency over animal healthcare.

*“I try and encourage people to do something rather than nothing… … and what we always said to start with was just put arnica in one pocket and aconite in the other one, cheap little squirt things, and if you walk round and you see a sick animal try it and see what happens”* – Participant 11 (Organic dairy farmer) [Interview excerpt].

Farmers rely on their own ideas of what early signs of disease are in certain animals. If an animal ‘is not quite right’ or is ‘a bit off’, a farmer might administer CAM products to prevent the development of something more severe.

*“…that’s what’s so important about having the box of remedies available or whatever because half of it is the fact that you actually do something rather than sitting there… if you have got, looking at an animal, and you think ‘it’s not quite right, I wonder what’s wrong with that? Oh, okay, it looks like it’s got a bad leg and maybe I’ll just give it some of this.”* - Participant 11 (Organic dairy farmer) [Interview excerpt].

### 3.4 Farming approaches associated with CAM use

Farmers who were: (1) working towards/currently part of production without antibiotic (PWAB) contracts (though few were already on these contracts at the time of interview); (2) currently or aiming to become certified organic farmers; (3) concerned about the overuse of antibiotics needed; or (4) wanted to reduce their use of antibiotics described using CAM to support this change. The organisations promoting PWAB and organic approaches promoted CAM approaches.

Farmers discussed milk contracts as an important influence on their use of antibiotics, which in turn influenced some farmers’ use of CAM. Several of the farmers were working under or towards OMSCo milk contracts. OMSCo is an organic dairy company that manages 65% of the UK’s organic milk supply and requires farmers to work towards reducing their AMU or take up a PWAB contract. Farmers often spoke of OMSCo in relation to promoting or providing access to some CAM courses.

*“Then, as I say, it was OMSCo who brought it [HAWL] down here originally for you [stockpeople] boys to go on it.”* - Participant 15 (Organic dairy farmer) [Interview excerpt].

One farmer suggested that to produce milk without the use of antibiotics, farmers have few options other than to use CAM to provide healthcare on farms.

*(I)* “*Is everyone that’s going zero antibiotics using these complementary alternative?*

*(R) They’ve got no choice have they? …So, you’ll find people that will think outside the box a lot more freely and probably forcing their vets to think outside the box as well. Because they know they cannot use antibiotics. Only as a last resort.”*- Participant 14 (Organic dairy farmer) [Interview excerpt].

Farmers referred to training and products that were promoted to them via the SA, to support them in reducing the use of some conventional medicines. This is via the RELACS project which aims to replace contentious inputs in organic farming systems as an EU-funded project (47). This project includes exploring the use of essential oils to support mastitis management and the use of naturally grown substances to reduce to use of anthelmintics. One farmer who was recruited onto the project stated that they were not yet able to fully engage with the project because their mastitis cases were already so low.

*“Yeah, which is part of the problem with this RELACS thing with this essential oil because we have not actually had a case of mastitis to treat in the last four or five months.”* - Participant 11 (Organic dairy farmer) [Interview excerpt].

One farmer was motivated to stop using antibiotics when they became organically certified. As such, this farmer felt more comfortable treating animals on a farm with little/no conventional veterinary medicines.

*“We transitioned [to an organic system] so in 2015 we stopped using antibiotics so from then that’s when the first years I’d say we had a lot of contact with him [vet] on a relatively regular basis. … Now we see the vet maybe four or five times a year or not even that and that’s usually during calving if there is a difficult calving or during the transition period for the cows. Apart from that we do not usually see him at all.”*– Participant 13 (Organic dairy farmer) [Interview excerpt].

## 4 Discussion

This paper describes the range of approaches, products, and principles that farmers consider to be CAM and their reasons for using CAM. This wide range of products and approaches included herbal remedies (e.g., commonly used topical udder creams including Uddermint®), homoeopathy, distant healing, environmental enrichment, observation methods, food products used for ingestion or external rubs, and shop-bought products. Not all participating farmers considered all of these products or approaches to be CAM. Some farmers used multiple approaches or products which they viewed as CAM whilst others just used Uddermint®, and did not regard this as CAM at all. CAM was thought of by some as a natural and holistic system approach, with products made with natural ingredients that supported animals’ natural healing processes. CAM was sometimes described as a logical approach and sometimes as magic. Farmers’ main motivation for using CAM was a desire to do something to support the health of their animals when other approaches were not available or appropriate. In using CAM, they were performing an act of care for their animals and fulfilling their responsibilities to look after their animals. The use of CAM was linked to organic farming and PWAB contracts as both restrict antibiotic use, which meant that some farmers were trying different approaches to supporting herd health.

CAM use is a polarized and controversial topic but continues to be practiced by farmers globally (1–3) and as this study has demonstrated, in the UK. The discussion about CAM in the veterinary literature often focuses on homoeopathy and the perceived limited evidence to support its use (48). However, this study found that farmers have a much broader idea of CAM that includes a wide range of approaches, products, and principles. Hellec et al. (49) found that farmers who also used a broad range of CAM adopted these therapies as part of a holistic approach that combined preventive and curative treatments that were grounded in paying close attention to animal health. This study found that these farmers were using CAM products and approaches to support both prevention and management of animal health issues in ways that were mainly complementary and sometimes (usually when conventional medicines were not available or appropriate) alternatives.

Defining what CAM is and includes can be challenging. The term ‘CAM’ is a political and social construction (55), often defined in terms of what it is not, as in the BVA (50) definition *‘treatments that fall outside of mainstream veterinary care’*. Hellec et al. (49) posited a difference between using complementary medicine and alternative medicine (also acknowledged by the BVA), in that ‘complementary’ seeks to support the use of conventional medicines whilst ‘alternative’ might suggest that therapies that are used to replace conventional medicine. However, farmers in this study rarely made this distinction, applying the general label ‘CAM’ to a wide range of products and approaches. CAM is an overly simplistic term for what is a complex range of practices and one that means different things to different stakeholders. It would be beneficial to consider what terminology would be appropriate to refer to this range of practices to improve both communication about such practices between farmers and their vets and wider discourses in the sector.

CAM was commonly referred to as a ‘natural’ approach to healthcare, either in the context of using natural substances, administering CAM in a natural way (e.g., ingestion as opposed to injection), or supporting an animal’s natural healing process. This association between ‘naturalness’ and CAM has also been observed in the human context and the idea that CAM is natural and therefore a safer approach to health is a common view amongst those who use CAM (51, 52). Wilcox et al. (52) found that parents viewed CAM to be an acceptable option for the treatment of children’s acute respiratory infections in part due to the belief that it is natural and therefore probably safer. Tangkiatkumjai et al. (51) reviewed 231 publications from 51 countries and noted that 8.7% of included publications noted natural-ness to be a factor that influences CAM use. Given that dairy farmers produce food for human consumption, there may be a desire to avoid the perceived risks of conventional medicine when caring for animals whose products will be consumed as food. Therefore, there is some evidence to suggest that farmers who were motivated to approach animal health management in a natural way were therefore more likely to explore CAM approaches.

This study found that CAM was sometimes used when farmers were trying to reduce the use of antibiotics, usually to achieve organic or PWAB certification. This aligns with a previous study that found that both conventional and organic dairy farmers in France were motivated to use CAM to enable them to provide care for animals that were not based on antibiotics or conventional veterinary treatment (49). This is not to suggest that CAM specifically influences farmers’ antimicrobial use, but rather that farmers are motivated to reduce antibiotic use for a variety of reasons, and that CAM is used to support them in doing so. This potentially useful contribution of CAM to supporting reduced antibiotic use has important implications for policymakers and the scientific community in the fight against the global health challenge of antimicrobial resistance. However, there remains the difficulty of squaring CAM use with EBVM from a veterinary perspective, which is made more difficult by the polarised debates and contention surrounding the evidence for homoeopathy specifically.

Official support and guidance surrounding CAM use was a subject of some discussion amongst all participants. Some farmers referred to the organic guidelines which state “*when treating you must use phytotherapeutic and homeopathic products and the trace elements, vitamins and minerals listed in standard 3.10.14 in preference to chemically-synthesised allopathic veterinary treatment”* [SA (53)]. This was in contrast with the RCVS statement which stated to vets, that CAM without a clear evidence base should not be used in place of effective conventional medicines. There was little evidence from this study to suggest that farmers would withhold or delay conventional treatment for animals. However, they were aware that CAM use was contentious and potentially disapproved of by the veterinary profession, which meant they were often reluctant to discuss their CAM use with their vet. This needs to be addressed so that at the very least, farmers can discuss their CAM use with their vet, preferably in such a way that organic farmers can abide by the organic guidelines (consult their vet when using CAM) and veterinary surgeons can support them in doing so without compromising their own professional practice (i.e., abiding by the RCVS guidelines).

## 5 Strengths and limitations

This is the first study to explore CAM use on dairy farms in a UK context. The qualitative approach was essential for the exploratory aims and eliciting the wide range of products and approaches that were included in farmers’ ideas of CAM and may not have been anticipated. It is possible that recall failure may mean that not all CAM products or approaches were remembered during interviews. It is also possible that participating farmers may have provided accounts that they felt the interviewer would prefer. Efforts were implemented to mitigate this possibility, including ethnographic observations whereby farm visits yielded further information to allow for comparison between what farmers described in interviews and what they stored on farms. Farmers were also informed that there were no ‘correct’ answers and that this study was entirely explorative with the aim of understanding their perspectives and experiences. A reflexive approach was implemented during data collection. For example, participants were made aware that the researcher (KC) was not a vet because it became clear that some farmers were less open to discussing CAM use with vets. Since recruitment materials stated the aim was to explore CAM use on farms, this may have resulted in participants who held a particular interest in this topic. Interpretation of these results was done whilst remaining mindful of these issues.

## 6 Conclusion

Farmers’ conceptualization of CAM varies and can include a very wide range of products and practices, the use of which is motivated mainly by their desire to provide care for their animals. It is proposed that CAM use should be acknowledged and discussed between all stakeholders, including vets, farmers, and CAM advisors. More open discussion surrounding the use of CAM on dairy farms would support better collaboration between dairy farmers their vets and other stakeholders in support of animal health. Vets, advisors, and farmers could engage in communities of practice and collaborative approaches to developing animal health management strategies that incorporate the safe and responsible use of CAM. This would support the study towards co-produced herd health plans that are farm-specific and acknowledge farmers’ own goals and values. This approach appears to have positive influences on farmers’ motivation to adhere to treatment and reduce unnecessary antibiotic use; yielding more positive animal health management outcomes.

It is also recommended that an understanding of CAM that reflects the reality of dairy farms is promoted within the veterinary sector. In the BVA position statement, CAM was essentially equated with homoeopathy and excluded from EBVM but this does not acknowledge the widely used products such as udder creams/oils or broader approaches such as animal observations or environmental enrichment, which some farmers consider CAM. There may be a benefit to discussing specific CAM products and practices by name, rather than using the term CAM more broadly, to avoid any confusion or negative associations with the term CAM. It is also important to acknowledge the potentially positive impacts of CAM, for example where it supports reduced use of antibiotics. Research into how CAM practices influence animal housing, management, and interactions should be conducted to identify potential positive practices resulting from farmer enthusiasm and contribute to the One Health approach for antimicrobial resistance impact reduction. This study shows that there is a need to find a way to allow farmers and vets to discuss CAM use on farms and this probably requires different terminology and a change in how CAM is viewed by the sector.

## Data Availability

The datasets presented in this article are not readily available because data generated from interviews with farmers and farm visits are confidential data. Requests to access the datasets should be directed to kayleigh.crouch@bristol.ac.uk.
