# Supplementary material for: What do complementary and alternative medicines mean to UK dairy farmers and how do they use them?
Source: Front Vet Sci. 2025 Feb 26;12:1504777. doi: 10.3389/fvets.2025.1504777 (PMC11897491; doi:10.3389/fvets.2025.1504777)

Supplementary Material

# Appendices

Appendix A: An exhaustive list of products and approaches used by farmers as part of their overall CAM approach. Details of which participating farmers engaged with each approach and what aspect of animal health they were used for.

| **CAM category** | **CAM/Approach** | **Used/stored for** | **Presented as CAM by** |
| --- | --- | --- | --- |
| Distant healing | Dowsing: In the form of 'distant' healing where they dowse for a homeopathic remedy using a pendulum and then send it on a Sulis machine. This sends the vibrations to the location of the intended to heal. | -Healing animals [and people] with positive vibrations | Farmer 15, Farmer 16, Farmer 17 |
| Homeopathic products | Calc Fluor | -Mastitis | Farmer 11, Farmer 15, Farmer 16, Farmer 17 |
|  | Calc Phos | -Delayed development  -Imperfect growth  -Inherited weakness  -Young calves | Farmer 15, Farmer 16, Farmer 17 |
|  | Calc Carb | -Manage weight and lethargic behaviour  -Support animals with anxious feelings  -Chest problems  -Joint and bone problems  -Urogenital problems  -Lethargic behaviours | Farmer 15, Farmer 16, Farmer 17 |
|  | Hepar Sulph | -Mastitis | Farmer 11, Farmer 15, Farmer 16, Farmer 17, Farmer 23 |
|  | Arnica | -Pain  -Arthritic/joint pain  -Throat soreness  -Bruising | Farmer 4, Farmer 10, Farmer 11, Farmer 15, Farmer 16, Farmer 17, Farmer 23 |
|  | Aconite | -Fear  -shock  -stress | Farmer 4, Farmer 11, Farmer 15, Farmer 16, Farmer 17, Farmer 23 |
|  | Hypericum/Calendula | -Injuries  -Cuts  -Abrasions | Farmer 4, Farmer 11, Farmer 15, Farmer 16, Farmer 17, Farmer 23 |
|  | Veratrum | -Cold sweats  -Body weakness  -Collapse | Farmer 11 |
|  | Pyrogen | -High fever  -Septicaemia  -Necrosis  -Retained Placenta | Farmer 11 |
|  | Ruta | -Sprains  -Muscle, ligament, and tendon strain | Farmer 11 |
|  | Ledum | -Insect bites  -Swelling  -Bruises  -Stings  -Puncture wounds | Farmer 11 |
|  | Conium | -Milk fever,  -E.coli Mastitis,  -Muscular Paralysis | Farmer 11 |
|  | Carbo veg (Corpse Revivers) | -Collapse  -Cold body  -Body weakness | Farmer 11, Farmer 15, Farmer 16, Farmer 17, Farmer 23 |
|  | Ignatia | -Depression  -Anxiety  -‘Go to remedy for cows mooing a lot’  -‘For anyone who is upset if you take their calf away’ | Farmer 11, Farmer 15, Farmer 16, Farmer 17, Farmer 23, Farmer 24 |
|  | Dulcamara | -Joint pain | Farmer 11 |
|  | Nux Vomica | -‘Off food’  -Digestive disturbance  -‘For the cows that have just calved and need to be picked up a little’  -‘For anyone who just ate too much cake’  -‘If someone is kicking up at their stomach’ | Farmer 11, Farmer 15, Farmer 16, Farmer 17, Farmer 23, Farmer 24 |
|  | Bellis Perennis | -Injury to trunk and deeper tissue (especially if stiff or cold develops)  -If arnica didn't have an effect | Farmer 15, Farmer 16, Farmer 17 |
|  | Antim Tart | -Maintain health  -Maintain respiratory system  - Pneumonia in calves  -Rattling breath  -Cough  -Tiredness | Farmer 15, Farmer 16, Farmer 17, Farmer 23 |
|  | Belladonna | -Milk fever  -Mastitis  -Restless cows  -Throwing heads around  -May also have fever | Farmer 15, Farmer 16, Farmer 17, Farmer 23 |
|  | Lychopodium | -Bloat  -digestive discomfort | Farmer 15, Farmer 16, Farmer 17 |
|  | Graphites | -Bloat  -Displace Abomasum  -Digestive discomfort | Farmer 15, Farmer 16, Farmer 17, Farmer 23 |
|  | Euphrasia | - Build up of mucous in airways and mucous membranes  -Allergies (particularly in eyes) | Farmer 15, Farmer 16, Farmer 17 |
|  | Conium | -Rigid animal  -Hard glandular swellings  -Tumours  -Support reproduction  -Encourage reproductive behaviours | Farmer 15, Farmer 16, Farmer 17 |
|  | Colchicum | -Digestive discomfort  -Build up of digestive gas | Farmer 15, Farmer 16, Farmer 17 |
|  | Kreosotium | -sickness  -Nausea | Farmer 15, Farmer 16, Farmer 17 |
|  | Lachesis | -Aggressive individuals  -Throat soreness (left side)  -Headaches  -Respiratory problems  -Hormonal difficulties  -Sensitivity to heat | Farmer 15, Farmer 16, Farmer 17 |
|  | Merc Corr | -Coccidiosis  -Intestinal troubles | Farmer 15, Farmer 16, Farmer 17 |
|  | Merc Soil | -Maintain general health/immune function | Farmer 15, Farmer 16, Farmer 17 |
|  | Phytolacca | -Mastitis  -Teat inflammation | Farmer 15, Farmer 16, Farmer 17, Farmer 23 |
|  | Podophyllum | -Cryptosporidium  -Enteritis  -Gastrointestinal upsets  -Rectal prolapse  -Uterus prolapse | Farmer 15, Farmer 16, Farmer 17 |
|  | Sabina | -Reproductive success  -Supporting calving  -Supporting pregnancy | Farmer 15, Farmer 16, Farmer 17, Farmer 23 |
|  | Sepia | -Reproductive health  -Reproductive support | Farmer 15, Farmer 16, Farmer 17, Farmer 23 |
|  | Sillica | -Calf sickness/vomiting | Farmer 15, Farmer 16, Farmer 17, Farmer 23 |
|  | Leptosporosis [highly diluted bacteria] | -Leptosporosis | Farmer 15, Farmer 16, Farmer 17 |
|  | Caulophyllum | -Stress  -Ease of calving  -Ease of weaning | Farmer 11, Farmer 15, Farmer 16, Farmer 17, Farmer 23, Farmer 24 |
|  | Ipecac | -blood in milk | Farmer 15, Farmer 16, Farmer 17, Farmer 23 |
|  | Kali Carb | -Weakness/paralysis post birth | Farmer 23 |
|  | Kali Phos | -Stimulate milk let down | Farmer 23 |
|  | Coccus | -Coccidiosis | Farmer 23 |
|  | Natrum Mur | -Increase milk production | Farmer 23 |
|  | Urtica | -Improve rumen function in lactating cow | Farmer 23 |
|  | Colocynthis | -Dysmenorrhoea  -Toothache  -Emotional pain  -Limb pain  -Numbness in the leg  -'if this one doesn't work [nux vomica]’ | Farmer 23, Farmer 24 |
|  | Viburnum | -Reproductive support | Farmer 23 |
|  | Dioscorea | -Pain relief for colic  -Abdominal pain  -Pelvic pain (when worse if lying down) | Farmer 23 |
|  | Passiflora | -Sleeplessness  -Restlessness  -Nervous behaviours | Farmer 23 |
|  | Drosera | -Coughing with gagging/retching | Farmer 23 |
|  | Phosphorus | -Prolonged wound bleeding  -Nervous behaviours | Farmer 23 |
|  | Apis Mel | -Restlessness  -Inflammation | Farmer 23 |
|  | Ferrum Phos | -Inflammation  -Fatigue  -Weakness | Farmer 23 |
|  | Gelsemium | -Fever | Farmer 23 |
|  | Pulsatilla | -Weaning/separation | Farmer 23 |
|  | Arsen Alb | -Food poisoning  -Nervous  -Lethargic | Farmer 23 |
|  | Rhus tox | -Rash  -Skin irritations | Farmer 23 |
|  | Staphisagria | -‘If you've got that feeling of being cut with a sharp knife’  -Post C-section recovery | Farmer 24 |
|  | Chamomilla | Mastitis | Farmer 23 |
| Pharmacies pre-made homeopathic kits | Pre-made kit: Ainsworth’s Bovine Homeopathic Veterinary Medicines |  | Farmer 15, Farmer 16, Farmer 17 |
|  | Pre-made kit: Crossgates bioenergetics Ltd Natural Animal Health Kit |  | Farmer 11, Farmer 15, Farmer 16, Farmer 17 |
|  | Pre-Made kit: Mother and children [formative years pregnancy- Age 7] |  | Farmer 23 |
| Bioresesonance Services and Homeopathic Products Developed | Herd specific stress/milk quality remedy (Crossgates bioresonsance service) | -Stress  -Milk quality | Farmer 11 |
|  | Ringworm remedy (Crossgates bioresonance service) | -Ringworm | Farmer 11 |
|  | Sepia mix (Crossgates bioresonance service) | -Nausea and vomiting for cows in calf | Farmer 11 |
| Holly Bush | Holly hung up in the parlour | -Ringworm | Farmer 6, Farmer 7, Farmer 12 |
| External Herbal Rubs | Comfrey | -Bruising | Farmer 15, Farmer 16, Farmer 17 |
|  | Calendula | -Abdominal discomfort post calving | Farmer 15, Farmer 16, Farmer 17 |
|  | Herbal salves – training in how to make own preparations with WHAg herbalist | -Mastitis  -Pneumonia  -Digestive functioning  -Worm control | Farmer 15, Farmer 16, Farmer 17 |
|  | Golden balm | -Mastitis  -Back soreness | Farmer 15, Farmer 16, Farmer 17 |
| Herbal Essential Oils | Lavender oil pessary | -Metritis | Farmer 3 |
|  | Citronella and lavender | -Calming cows in stressful situations | Farmer 24 |
|  | Unspecified | -Mastitis control  -Digestive function | Farmer 11, Farmer 14, Farmer 21, Farmer 22 |
|  | Avena Masticare (essential oil based rub) | -Mastitis control  -Mastitis prevention | Farmer 14 |
| Products Ingested for Gut Health | Kefir | -Supporting calf growth and digestive health | Farmer 15, Farmer 16, Farmer 17 |
|  | Apple cider vinegar | -Maintain healthy digestion/gut health  -Drench for cows with mastitis | Farmer 15, Farmer 16, Farmer 17, Farmer 21 |
|  | Sea Solids | -Gut health maintenance | -Farmer 15, Farmer 16, Farmer 17 |
|  | GALLIC Pruex: Complementary feed supplement for ruminants | -Maintain gut health | Farmer 15, Farmer 16, Farmer 17, Farmer 21, Farmer 22 |
|  | Garlic during drying off [in feed] | -Mastitis prevention | Farmer 14, Farmer 15, Farmer 16, Farmer 17 |
| Products Used as Internal Washouts | Coffee | -Displaced abomasum | Farmer 8, Farmer 19, Farmer 22 |
|  | Utresept | -“Uterine wash out which has yukka in to make it soapy and then citric acid and orange gets rid of the bugs or helps with cleansing and retained placentas” | Farmer 24 |
| Products Used as External Rubs | Manuka honey | -Wound management  -Healing sores | Farmer 2, farmer 3, Farmer 13, Farmer 14, Farmer 15, Farmer 16, Farmer 17, Farmer 19, Farmer 21, Farmer 22 |
| Products Used as External Washouts/External Application | Skin-so-soft (Avon) moisturiser and cold tea | -Fly repellent  -Stress management | Farmer 20 |
|  | Stinky stuff: Natural balm which soothes irritated skin and contains Nigella sativa, cocos nucifera, butyrospermum, parkii butter, euphorbia cerifera cera wax. | -Foot health maintenance | Farmer 20 |
|  | Epsom salts | -Digital dermatitis | Farmer 6, Farmer 7 |
|  | Listerine udder washout | -Mastitis prevention | Farmer 10 |
|  | Germolene | -Numbing painful mastitis | Farmer 8 |
|  | Stockholm foot tar/Pine resin | -Mastitis  -Fly repellent  -Wound barrier | Farmer 10, Farmer 11, Farmer 18 |
|  | Coconut oil | -Ant inflammatory  -Mastitis prevention | Farmer 15, Farmer 16, Farmer 17, Farmer 21 |
|  | Peanut oil | -Fly repellent  -Bloat | Farmer 19 |
|  | Uddermint® | -Uddercream liniment containing 35% Japanese Peppermint Oil | All participating farmers |
| Observational Tools | Constitutional element of homeopathy | -Monitoring animal health and disease  -Considering the behaviour and character of the animal to identify the most appropriate remedy | Farmer 3, Farmer 5, Farmer 10, Farmer 11, Farmer 15, Farmer 16, Farmer 17 |
|  | Obsalim® | -Structured observations using cards to draw associations between health and nutrition | Farmer 4, Farmer 11, Farmer 18, Farmer 23, Farmer 24 |
|  | Technology (senshub, ear tags, moo meters) |  | Farmer 1, Farmer 2, Farmer 3, Farmer 5, Farmer 8, Farmer 22 |
|  | Robotic milking | -Monitor animal health during milking [somatic cell count and temperature of udder]  -Controlling/prevent mastitis  -Inform the use of other CAM products | Farmer 9, Farmer, 14, Farmer 19, Farmer 22 |
| Enrichment | Occupational Enrichment: Used to mentally stimulate animals | -Traffic cone/novel objects in enclosure | Farmer 2, Farmer 3 |
|  | Mechanical cow brushes | -Maintain healthy coats  -Improve quality of life for cows | Farmer 1, Farmer 2, Farmer 3, Farmer 6, Farmer 7, Farmer 10, Farmer 19, Farmer 21, Farmer 22 |
|  | Human animal interaction/calm talking/singing | -Reduce stress at handling to maintain a well-functioning immune system | Farmer 1, Farmer 2, Farmer 3, Farmer 5, Farmer 19, Farmer 21 |

Appendix B: A topic guide used to support the researcher during semi-structured interviews. This was used only as a guide to ensure that relevant topics were covered during each of the interviews.

**General Introductory Questions**

How did you get into farming?

What drove you to stay within farming?

**Initial CAM Use Questions**

What comes to mind when you think of CAM?

What CAM do/have you used?

How did you begin using/become interested in CAM?

Do you know others who use CAM?

Have you/anyone you know ever used CAM for your own health? Own pets health?

What is your perception of CAM? Does CAM have a reputation? How would you describe how its’ viewed by the farming community?

**CAM Application**

Can you describe to me how you decide what treatment to use?

Can you tell me about any training you have had in using CAM?

Can you tell me about conversations you have with your vet about CAM?

When would you decide to use CAM (preventative, alongside prescribed Veterinary Medicine, last resort?)

**Outcomes of CAM Approaches**

What are the benefits of using CAM?

What is your main reason for using CAM?

Which of these would you consider most fundamental in your using CAM?

Probing Questions: *use of biomedicine/ vet approved mediciness/ antibiotics*

**Practice-Based Effects**

Do you think that using CAM effects the way you (or others) interact with the animals on farm? (probing; time, housing, talking to animals, massage, observation of animals)

What are your experiences and opinions of a more digital approach?

**Concluding Questions**

How would you describe your approach to farming?

*What would you say are your main priorities as a farmer?*

Is there anything that you would like to add?

Appendix C: Participant information sheet for farmer semi-structured interviews, providing a background to the research study before farmers gave informed consent.


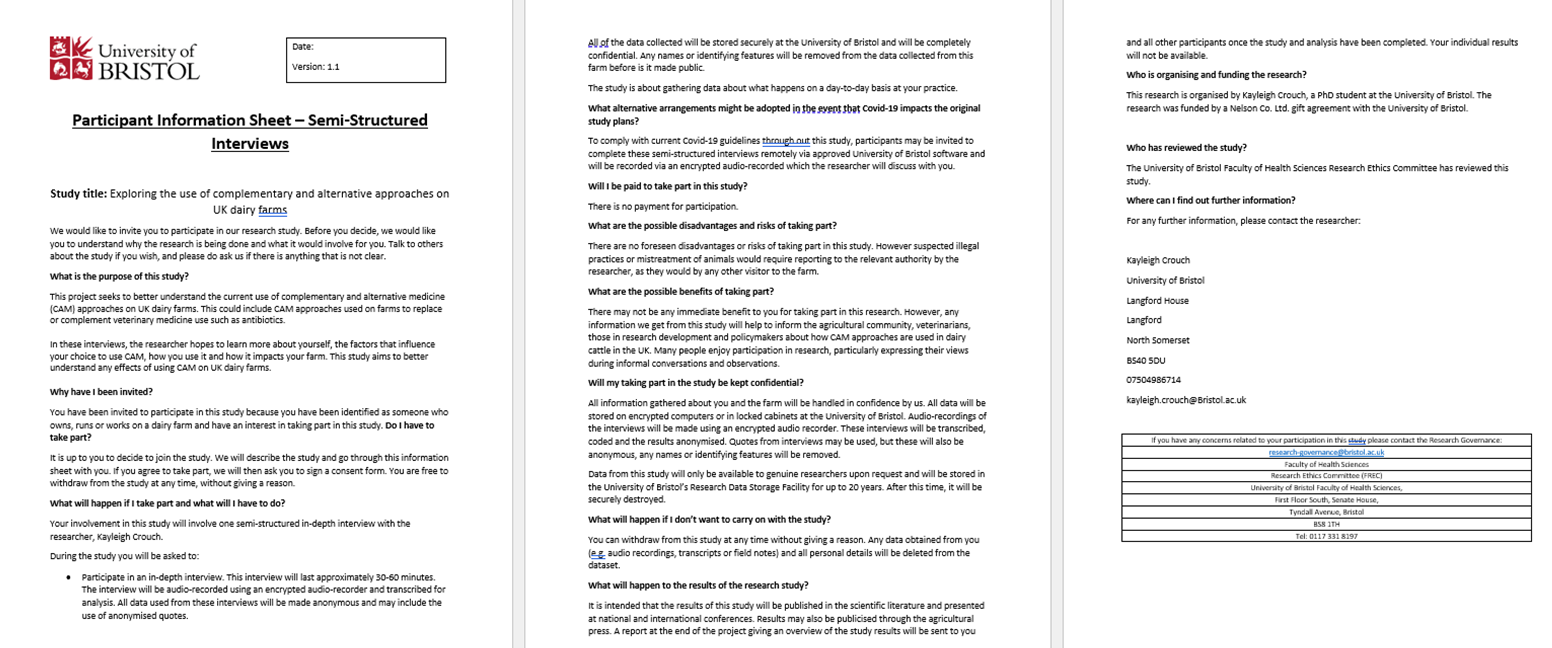


Appendix D: Participant information sheet for farm visits and non-participant observations, providing a background to the research study before farmers gave informed consent.


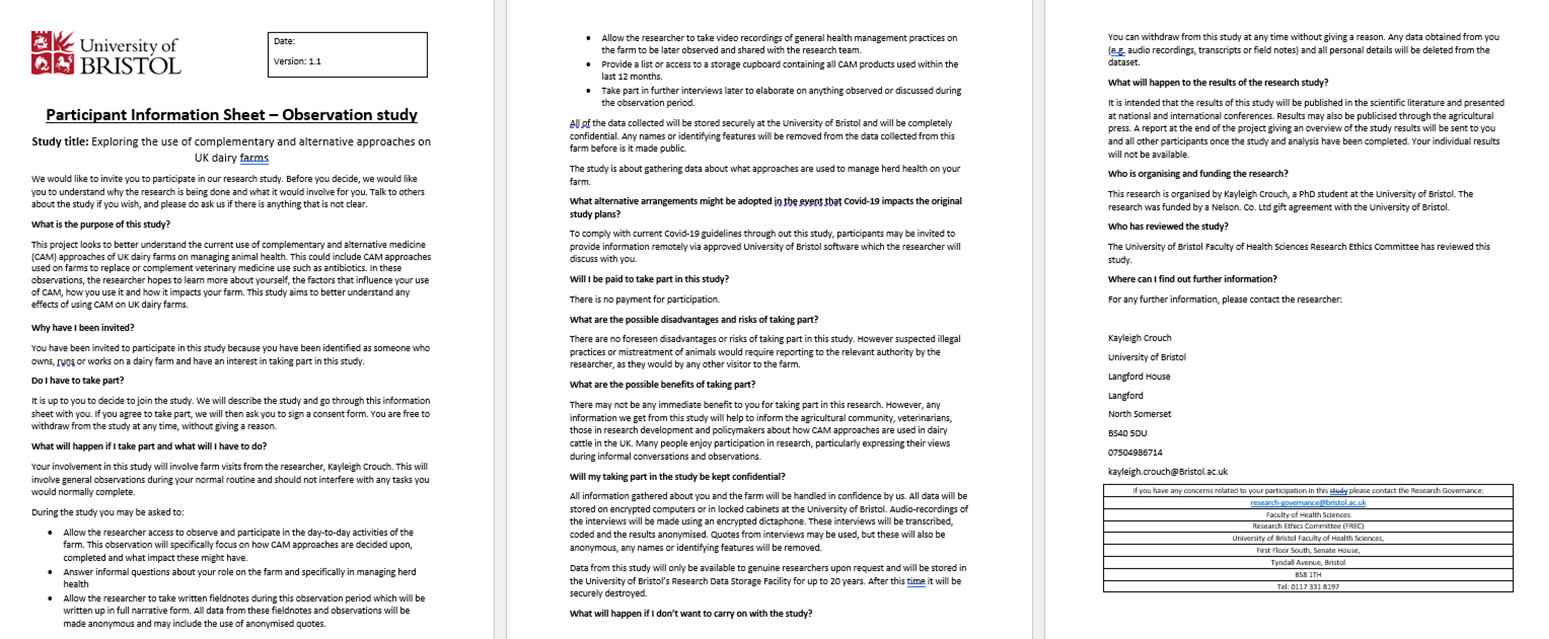


Appendix E: The form used to obtain informed consent from farmers during semi-structured interviews.


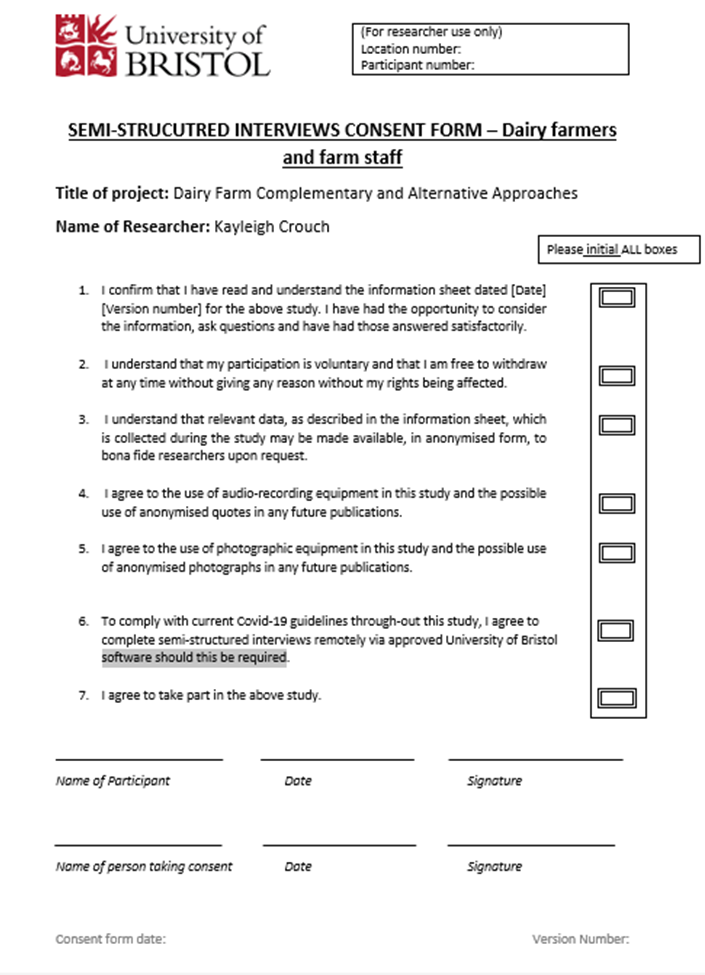


Appendix F: The form used to obtain informed consent from farmers during farm visits and non-participant observation.


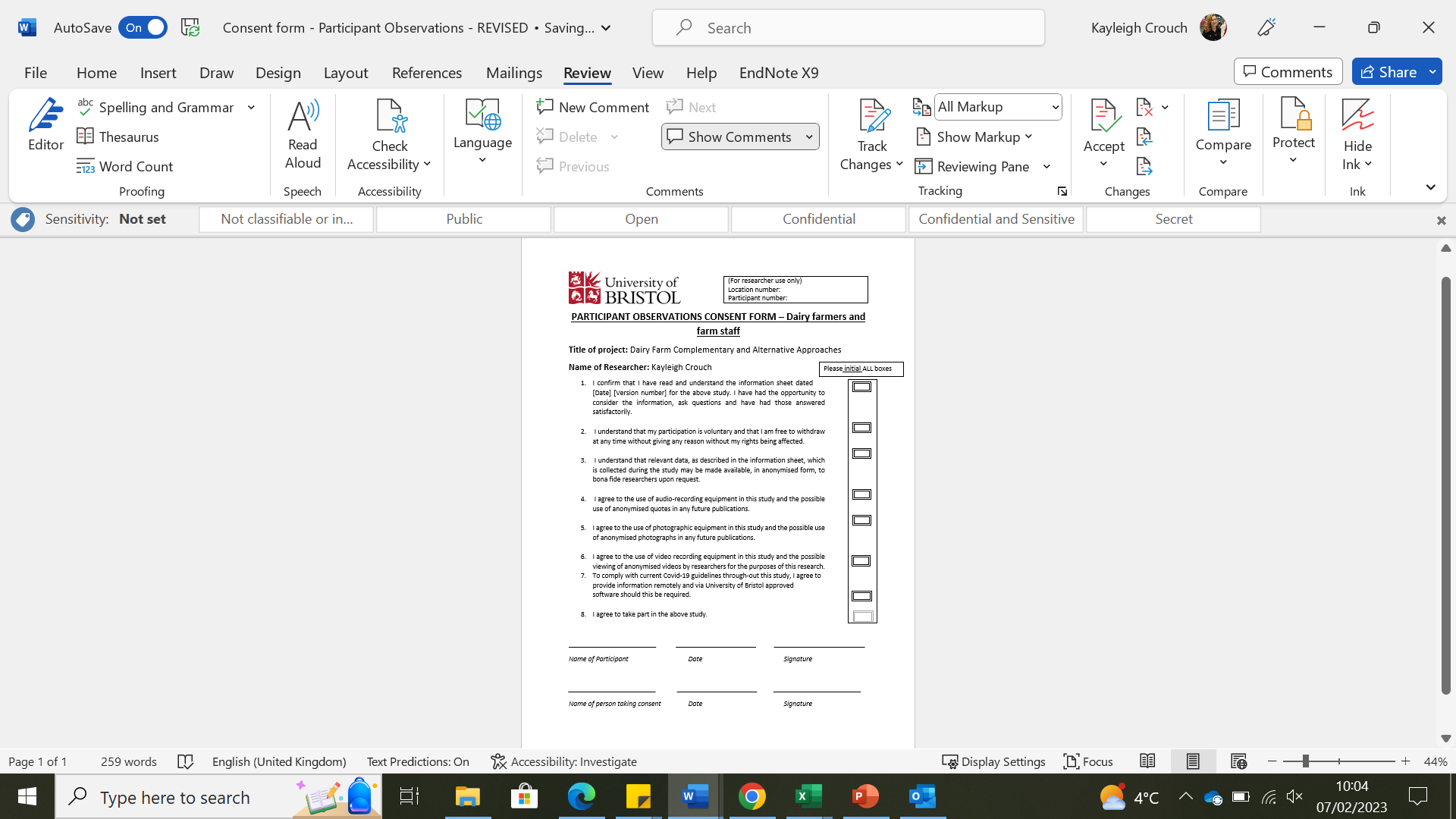

Supplement: Supplementary file 1 [file Table_1.docx]
